# Telepathology and Mobile Health System for Province-Wide Pathology Consultation in Henan, China: Retrospective Evaluation Study

**DOI:** 10.2196/75172

**Published:** 2026-03-09

**Authors:** Jinming Shi, Ming Ye, Dongxu Sun, Xianying He, Yaoen Lu, Linlin Wang, Haotian Chen, Wenchao Wang, Jie Zhao, Fangfang Cui

**Affiliations:** 1 National Engineering Laboratory for Internet Medical Systems and Applications First Affiliated Hospital of Zhengzhou University Zhengzhou, Henan China; 2 Shanghai Artificial Intelligence Laboratory Shanghai China; 3 Institute of Intelligent Medicine Henan Academy of Innovations in Medical Sciences Zhengzhou, Henan China

**Keywords:** telepathology, diagnostic equity, digital pathology, remote diagnostics, rural health, health systems innovation

## Abstract

**Background:**

Telepathology has emerged as a transformative digital health solution to address the global shortage of pathologists and the unequal distribution of diagnostic services, particularly in underserved and rural areas. In Henan Province, China, high diagnostic demand, rapid population growth, and limited pathology expertise exacerbate regional health care inequities, leading to delayed diagnoses and restricted access to specialist care.

**Objective:**

This study aimed to design, implement, and evaluate a province-wide telepathology system integrating web and mobile platforms to enhance diagnostic quality, efficiency, and equitable access across health care tiers.

**Methods:**

We conducted a retrospective, multicenter observational study using deidentified data from 120 health care institutions between 2016 and 2024. The system used a 3-tier architecture with virtual private network–secured transmission and a Browser-Server framework, supporting standardized whole-slide image acquisition, remote review, and reporting via web interfaces and a WeChat (Tencent) mini-program. System performance was assessed by consultation volume, turnaround time, concurrency, and diagnostic concordance in a subset of 1027 cases with paired tertiary-hospital expert diagnoses. Economic impact was estimated using previously published per-case savings, reflecting patient travel and ancillary cost reductions. Additional assessments included workflow integration, mobile platform use, and system stability under peak load.

**Results:**

Over 8 years, the network processed 72,916 consultations encompassing 355,104 whole-slide images, supporting 220-300 concurrent users with stable performance. Median turnaround time was 10.06 (IQR 1.63-29.10) hours, with 96.41% (70,298/72,916) of cases completed within 72 hours. County-level hospitals contributed 77.63% (56,603/72,916) of consultations, demonstrating substantial engagement from lower-tier institutions. In the diagnostic subset, originating-site preliminary classifications achieved 0.90 sensitivity and 0.75 specificity relative to expert reference diagnoses, with 17.2% discordance corrected through remote expert review. Estimated annual direct cost savings ranged from US $0.14 to $0.63 million. Mobile-enabled access facilitated remote review and reporting without compromising data security, supporting integration into routine clinical workflows across diverse hospital settings.

**Conclusions:**

The Henan Province telepathology system demonstrates that a centrally coordinated, scalable digital health platform can improve diagnostic efficiency, quality, and equity in resource-constrained settings. High county-level hospital use highlights its potential to reduce geographic and structural diagnostic inequities. Future work should explore formal cost-effectiveness evaluation, artificial intelligence–assisted diagnostic support, and cross-regional interoperability to enable broader adoption and sustainable integration into health care systems.

## Introduction

The digitization of pathology workflows has revolutionized diagnostic practices. High-throughput slide scanning and standardized data management have enabled unprecedented improvements in service accessibility and interinstitutional collaboration [1]. Digital pathology (DP) offers multiple benefits, including enhanced diagnostic accuracy, faster turnaround times (TAT), and increased efficiency in pathology workflows [2-5]. However, translating these technological advancements into equitable real-world health care delivery remains constrained by 2 systemic barriers: the geographic concentration of pathology expertise and the lack of scalable infrastructure to support cross-regional coordination [6]. In Henan Province, China, a region with a population exceeding 100 million and reporting approximately 279,740 new cancer cases annually [7], these challenges are particularly pronounced. The province faces a critical shortage of pathologists, who constitute only 0.4% of the medical workforce [8,9]. This shortage leads to significant delays in diagnosis, especially in rural and underserved areas, where patients often face prolonged wait times for pathology reports. These diagnostic delays directly contribute to adverse patient outcomes, including delayed treatment initiation and reduced survival rates, while exacerbating health care inefficiencies [7,10]. These diagnostic delays are symptomatic of deeper structural inequities in health care access between urban and rural populations.

Telepathology has emerged as a transformative solution to decentralize diagnostic services, offering a viable strategy to overcome these systemic barriers [11-14]. By leveraging digital slide scanning technologies [15], secure data transmission protocols [16], and remote diagnostic interfaces, telepathology enables cross-regional and cross-institutional collaboration among pathologists [17,18]. Numerous studies have demonstrated that telepathology systems can effectively reduce TAT, enhance diagnostic accuracy, and optimize resource distribution, making them valuable tools for both routine diagnostics and specialized consultations [19-24]. For instance, a telepathology diagnostic network established in Tanzania showed TAT performance comparable to conventional intraoperative consultations using optical microscopes [23]. Similarly, the Clinical Telepathology Service at the University of California, Los Angeles, demonstrated improved diagnostic efficiency and cost savings through its cloud-based infrastructure [25].

However, the scalability of telepathology systems remains a critical barrier to widespread adoption. A recent systematic review revealed that the majority of telepathology initiatives are limited to single institutions or pilot programs, with relatively few achieving province- or state-wide coverage [26]. This limitation arises from multiple technical and organizational challenges, including inconsistent standards for image acquisition, storage, and sharing, which hinder interoperability across regions and institutions [26]. Additionally, the large volumes of data generated by whole slide image (WSI) systems contribute to computational bottlenecks, complicating data processing and analysis [15,16]. The absence of tiered governance models, which are essential for coordinating multilevel health care systems, further constrains the effective management and deployment of telepathology solutions [27,28]. Moreover, without equitable deployment strategies, such innovations risk widening existing gaps in diagnostic access.

To address these challenges, this study presents the design, deployment, and evaluation of a province-wide telepathology system across 120 health care institutions in Henan Province, China. Enabled by dedicated funding from the Henan Provincial Health Commission, the system facilitated the deployment of DP scanners in county- and city-level hospitals. Led by the First Affiliated Hospital of Zhengzhou University, the largest medical institution in China, the system operates under a centralized framework for case management, expert consultation, and data integration, supported by a mobile-enabled interface to expand diagnostic access to remote and rural regions.

This study aims to (1) design and implement a scalable province-wide telepathology system to enhance equitable access to diagnostic services across health care tiers; (2) evaluate its performance in terms of diagnostic efficiency, accuracy, and economic impact (direct patient cost savings); (3) demonstrate the feasibility of large-scale telepathology expansion in resource-constrained settings; and (4) offer a scalable model that can be replicated in other health care systems facing similar challenges. These findings contribute to the growing body of evidence supporting digital health solutions in bridging health care gaps and optimizing pathology services worldwide.

## Methods

### Overview

This Methods section describes the study design, data sources, evaluation methods, and data protection measures used to assess a province-wide telepathology system.

### Study Design

This study adopted a multicenter retrospective observational design to evaluate the real-world implementation and performance of a province-wide telepathology system across 120 health care institutions in Henan, China. Routinely collected, deidentified system data generated between January 2016 and June 2024 were analyzed.

The evaluation comprised three components aligned with the study aims: (1) technical performance, assessed using Apache Benchmark stress testing to measure response time and concurrent user capacity; (2) operational use and efficiency, evaluated descriptively using system-generated metrics including consultation volume, contributing hospital level, and diagnostic TAT; and (3) diagnostic accuracy, evaluated in a retrospective subset of 1027 cases by comparing originating-site preliminary diagnoses with tertiary-hospital expert reference diagnoses recorded in the platform.

### Data Collection and Sources

All analyses used routinely collected, deidentified data automatically recorded by the telepathology platform between January 2016 and June 2024. System logs and administrative exports were used to summarize consultation volume, contributing hospital level, annual trends, and TAT, defined as the interval from WSI submission to report issuance.

For frozen-section consultations, the request-to-diagnosis time was summarized based on real-time notification and reporting timestamps recorded in the platform. Patient and diagnostic characteristics (age, sex, benign vs malignant classification, and specimen anatomical site) were obtained from system-generated pathology reports. Platform metadata and storage reports were used to describe supported WSI formats, per-slide file size range, and cumulative storage volume.

### Diagnostic Accuracy Assessment

Diagnostic accuracy was evaluated in a retrospective subset of 1027 telepathology consultations that had both (1) an originating-site preliminary diagnosis recorded at submission and (2) a corresponding tertiary-hospital expert diagnosis recorded in the platform for the same case. Cases with missing or noncomparable diagnostic categories were excluded. Diagnoses were dichotomized as malignant (positive) versus non-malignant (negative) according to the recorded report impression. The tertiary-hospital expert diagnosis served as the reference standard. Sensitivity, specificity, and overall agreement were calculated using a confusion matrix.

### Economic Impact Estimation

Per-case cost savings were not recalculated from primary economic data in this study. Instead, we adopted the previously published per-case savings estimate for the same provincial telepathology program (378.5 RMB per case; US $1=6.46 RMB) reported by He et al [10], which was based on local consultation fees and assumed travel and food expenses avoided when patients no longer needed to travel to tertiary hospitals for expert consultation. To derive an annual estimate, we multiplied the per-case savings (378.5 RMB; US $1=6.46 RMB) by the number of completed telepathology consultations in each calendar year (Table 1). All RMB values were converted to US dollars using the exchange rate reported in He et al [10].

**Table 1:** Annual volume of completed telepathology consultations by hospital level in a province-wide observational study conducted in Henan Province, China.

Hospital level	2016	2017	2018	2019	2020	2021	2022	2023	2024
City-level	240	657	1060	923	2100	3503	2970	2439	2421
County-level	2095	3673	6202	8317	7490	6536	6198	8267	7825

### System Architecture Design

To address the shortage of pathologists and the uneven distribution of diagnostic resources in Henan Province, China, we designed an integrated province-wide telepathology system optimized for scalability, diagnostic accuracy, and operational efficiency. The system follows a 3-tiered hospital architecture, consisting of tertiary-level hubs (provincial hospitals), secondary-level nodes (city-level hospitals), and primary-level health care institutions (county-level hospitals). This hierarchical structure enables standardized workflows for digital slide acquisition, data transmission, remote diagnosis, and reporting across 120 health care institutions, ensuring equitable access to high-quality pathology services.

**Figure 1:**
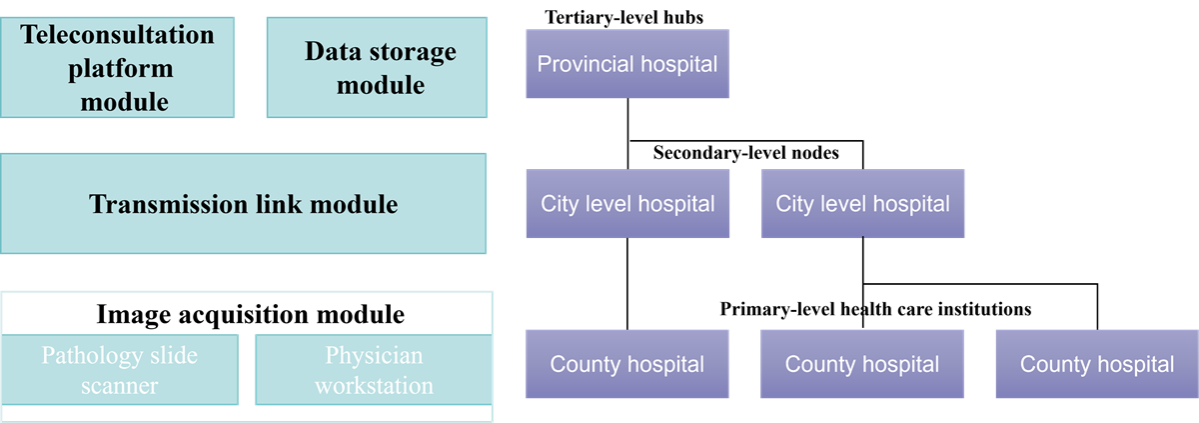
Architecture of the province-wide telepathology system evaluated in a retrospective multicenter observational study across 120 health care institutions in Henan Province, China.

The system adopts a Browser-Server architecture, chosen for its flexibility, ease of deployment, and compatibility with diverse IT infrastructures in both urban and rural health care settings. To support real-time access in resource-constrained environments, we incorporated both web-based platforms and mobile access via a WeChat mini-program. This mobile interface was particularly critical for ensuring remote diagnostic access in rural areas where conventional IT infrastructure is limited. The mini-program was selected due to its widespread adoption in China and seamless integration with existing health care communication workflows, eliminating the need for standalone app installations. The system consists of 4 core modules: image acquisition module, transmission link module, data storage module, and teleconsultation platform module. These modules are tightly integrated to support high-throughput data processing, secure transmission, and efficient telepathology consultations (Figure 1).

### Image Acquisition Module

This module digitizes traditional pathology slides into high-resolution WSIs and prepares them for secure transmission. Each participating hospital is equipped with (1) pathology slide scanner Model KF-PRO-005 (Konfoong Biotech International Co., Ltd.), capable of scanning slides within 40 seconds at 0.47 µm per pixel (20 magnification) and within 100 seconds at 0.5 µm per pixel (40 magnification); and (2) physician workstation powered by Intel Core i5 processors with 8 GB RAM, preloaded with K-Scanner 1.6.0.14 and K-Viewer 1.5.3.1 (Konfoong Bioinformation Tech Co, Ltd., Ningbo, China) for scanner control, image browsing, and management.

The scanned WSIs are processed using a multiresolution pyramid structure, optimizing image loading speed and reducing data size without compromising diagnostic quality.

In addition, a standardized scanning workflow and a 2-step image quality control (QC) procedure were applied across participating sites to ensure that WSIs met diagnostic requirements. Before scanning, slides were checked for correct labeling and basic preparation adequacy, and scanners were routinely calibrated according to the manufacturer’s recommendations. After scanning, technicians performed an on-screen QC review to confirm tissue coverage completeness, focus adequacy across the tissue area, and absence of major scanning artifacts (eg, excessive blur, stripe or banding, or large occlusions), as well as reasonable stain and color appearance. Slides failing QC were rescanned, and if persistent issues were attributed to slide preparation, the originating laboratory was notified for recutting or restaining. A second, preanalytical QC check was conducted by triage personnel during case allocation; cases with insufficient image quality were returned for resubmission prior to expert review.

### Transmission Link Module

**Figure 2:**
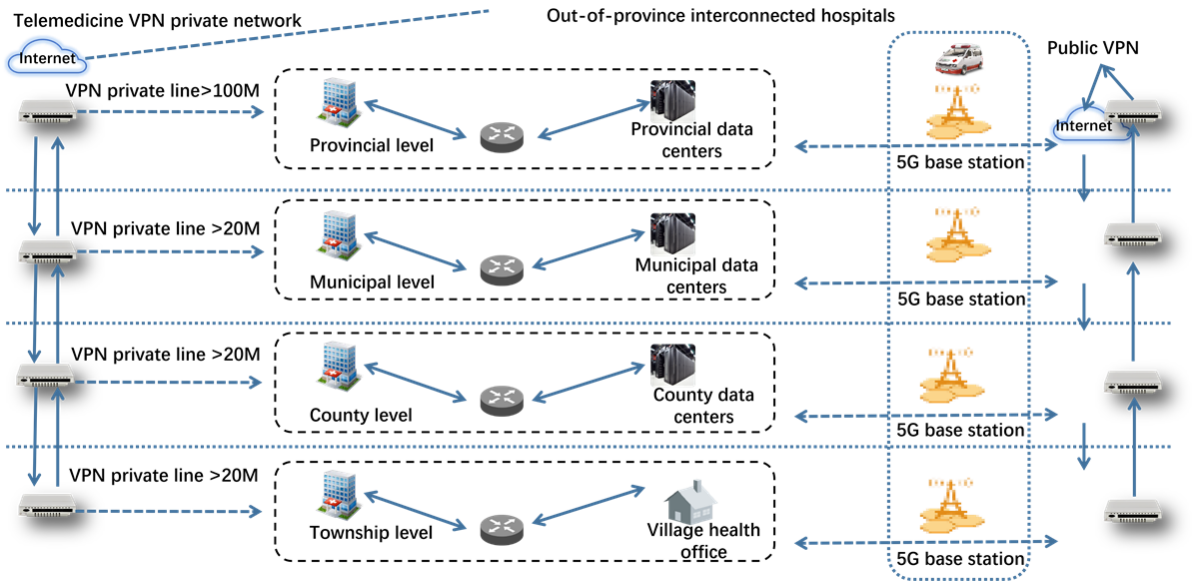
Architecture of the medical private network supporting province-wide telepathology consultations in Henan Province, China.

The transmission link module ensures that digitized pathology images can be transmitted from the applying hospital to higher-level hospitals. Considering data security and transmission rates, the system uses the 5G fixed-mobile convergence medical private network, previously established by the Henan Telemedicine Center of China and covering Henan Province, as the transmission link. Digitized pathology slides are scanned and uploaded to the central server for storage and diagnosis through this private network. The architecture of this private network is illustrated in Figure 2.

The telemedicine private network can be classified into wired transmission networks, 5G telemedical transmission networks, and Internet of Things transmission networks. The wired network is primarily used in fixed office scenarios and easily accessible medical institutions, such as large hospitals, for teleconsultations, telepathology, remote education, and applications in medical institutions at the county level and above. The 5G telemedical transmission network mainly supports dynamic scenarios, off-hospital scenes, and the access of medical institutions in remote areas, including remote ward rounds, remote emergency services, remote electrocardiography, remote ultrasound, and access for village and town health clinics. The construction and application of this private network provide effective network support for the transmission of remote pathological slides.

### Data Storage Module

The data storage module is designed to ensure the secure, scalable, and efficient storage of WSIs, diagnostic reports, and patient records. To enhance storage efficiency and fault tolerance, we use erasure coding technology, which works by dividing data into multiple fragments and generating parity blocks distributed across different storage nodes. In the event of data corruption or hardware failure, erasure coding enables data reconstruction from the remaining fragments, ensuring high availability and data integrity with minimal storage overhead [29].

For data backup, the system adopts a master-slave data synchronization strategy, implementing asynchronous replication to ensure the consistency and integrity of data. Additionally, binary log (Binlog) technology is used to record all database changes, allowing for rapid data recovery in the event of a system failure. The backup mechanism uses a mixed-based replication model, combining the advantages of statement-level and row-based replication, ensuring efficient and stable data backups.

### Teleconsultation Platform Module

#### Overview

**Figure 3:**
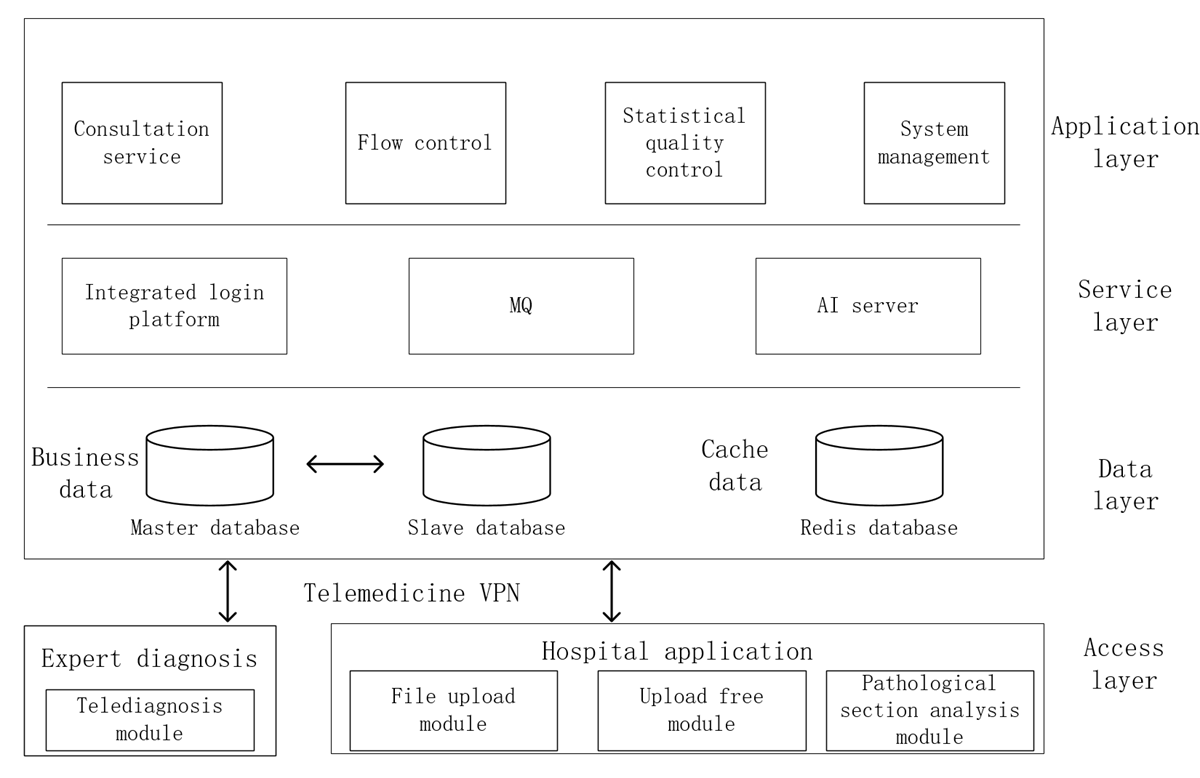
Software architecture of the teleconsultation platform used for province-wide telepathology services in Henan Province, China. AI: artificial intelligence; MQ: message queue; VPN: virtual private network.

The teleconsultation platform is the core of the system, supporting remote pathology consultations, diagnostic reporting, and interinstitutional case discussions. The software architecture (Figure 3) is organized into 4 layers.

#### Access Layer

The access layer connects requesting hospitals with expert terminals, enabling seamless interaction and workflow integration. Within this layer, the upload free module eliminates the need for manual file uploads by integrating directly with local DP scanners, ensuring automatic and efficient transmission of WSIs. Additionally, the Pathological Section Analysis Module performs preliminary analysis, including image quality assessment, segmentation, and lesion detection, aiding triage personnel in prioritizing cases. To optimize performance, WSI images undergo segmentation, compression, and pyramid layer processing, allowing for rapid loading and smooth scaling of high-definition images. Experts can access, analyze, and report cases either through a web browser or via the WeChat mini-program, enhancing flexibility and accessibility.

#### Data Layer

The data layer is responsible for managing both business-related data and cache data to ensure system stability and efficiency. Business data is stored within a MySQL relational database, maintaining structured and secure data management, while cache data, such as user authentication tokens and system configuration information, is stored using Redis, an in-memory database designed to facilitate high-speed data access and enhance system performance.

#### Service Layer

The service layer provides the system's core functionalities, encompassing consultation process management, diagnostic reporting, and QC statistics. To ensure seamless communication between different modules, message queue technology is implemented, allowing for asynchronous data transmission. This enables efficient handling of high-volume concurrent requests, ensuring that teleconsultation processes remain reliable and scalable without system bottlenecks.

#### Application Layer

The application layer provides various application functionalities and permissions tailored for triage personnel, system administrators, primary hospital doctors, and others, catering to their specific roles and access levels.

Data protection and privacy. To support province-wide deployment and mobile access, the platform implemented the following safeguards. The WeChat mini-program served only as a mobile access interface. WSIs and clinical records remained in the telemedicine center’s private cloud and were accessed through the secured telepathology platform via a dedicated application programming interface gateway (HTTPS with Transport Layer Security encryption, authentication, role-based access control, and an allowlist of permitted application programming interfaces). Access events were logged for audit and monitored with rate limiting when needed. The WeChat-based support chat group was limited to technical troubleshooting and operational coordination; no patient-identifiable information or diagnostic images were shared, and all clinical case review and reporting were performed within the secured telepathology platform.

### System Functionality Overview

#### Overview

During the functional design phase of the system, a multidisciplinary design discussion group was established to ensure that the system effectively met the needs of different stakeholders. This group consisted of 6 members, including 1 pathologist from the First Affiliated Hospital of Zhengzhou University, representing the requirements of tertiary hospitals, 2 pathologists from primary health care institutions who articulated user needs from a clinical perspective, 1 project manager overseeing system development, and 2 software developers responsible for technical implementation. To validate the system's diagnostic reliability, a clinical study was conducted in which pathologists compared WSI-based diagnoses with conventional glass slide diagnoses across various case types. Expert feedback was collected on image interpretability, usability, and diagnostic confidence using both the browser-based platform and the WeChat mini-program. The results demonstrated a high concordance between digital and conventional diagnoses, confirming the system's suitability for clinical application.

**Figure 4:**
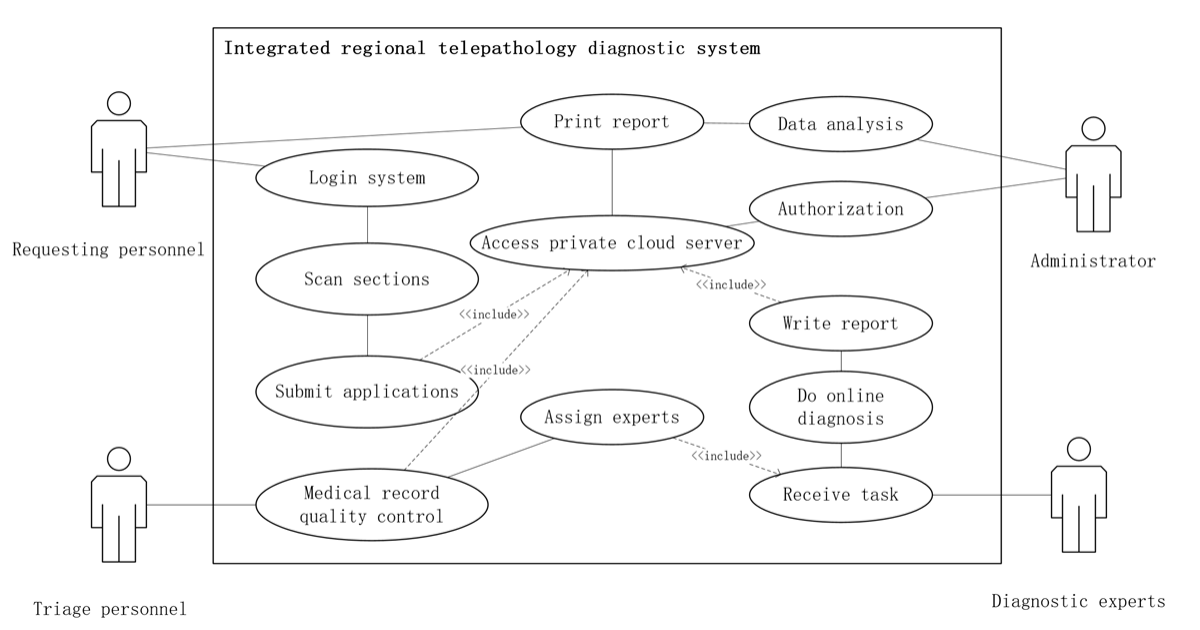
Use case diagram illustrating roles and interactions within the province-wide telepathology system in Henan Province, China.

Based on functional requirements, users were categorized into 4 distinct roles within the telepathology framework, ensuring an optimized workflow that facilitates seamless collaboration between requesting personnel, triage personnel, diagnostic experts, and system administrators (Figure 4).

#### Requesting Personnel

Primarily from county-level (primary) and city-level (secondary) hospitals, initiate telepathology processes by digitizing and scanning pathology slides, completing patient medical records, and submitting telepathology requests to higher-level institutions. This decentralized design enabled even low-resource county hospitals to initiate telepathology consultations independently, contributing to more equitable diagnostic access.

#### Triage Personnel

As pathologists within city-level (secondary) and provincial-level (tertiary) hospitals, they are responsible for preanalytical QC and case allocation. By leveraging their in-depth knowledge of their hospital's pathology experts and their areas of specialization, they ensure that each case is assigned to the most suitable specialist within the institution.

#### Diagnostic Experts

Consisting of pathologists within city-level and provincial-level hospitals, analyze the assigned digital slides and generate diagnostic reports, either providing a direct diagnosis or escalating particularly complex cases to a higher-level institution when necessary.

#### Administrators

They oversee the integrated telepathology system, managing user roles and permissions, ensuring data security, and maintaining system functionality to guarantee a seamless telepathology workflow.

### Operational Workflow and User Interaction

#### Overview

The integrated telepathology system is designed to streamline the end-to-end diagnostic workflow, encompassing 3 primary functionalities: application initiation, triage, and diagnostic review. These functionalities are supported by an intuitive user interface that facilitates efficient interaction between primary health care providers, triage coordinators, and diagnostic experts. This section provides a detailed description of the system’s operational flow, user roles, and key technical features.

#### Application Initiation Workflow

**Figure 5:**
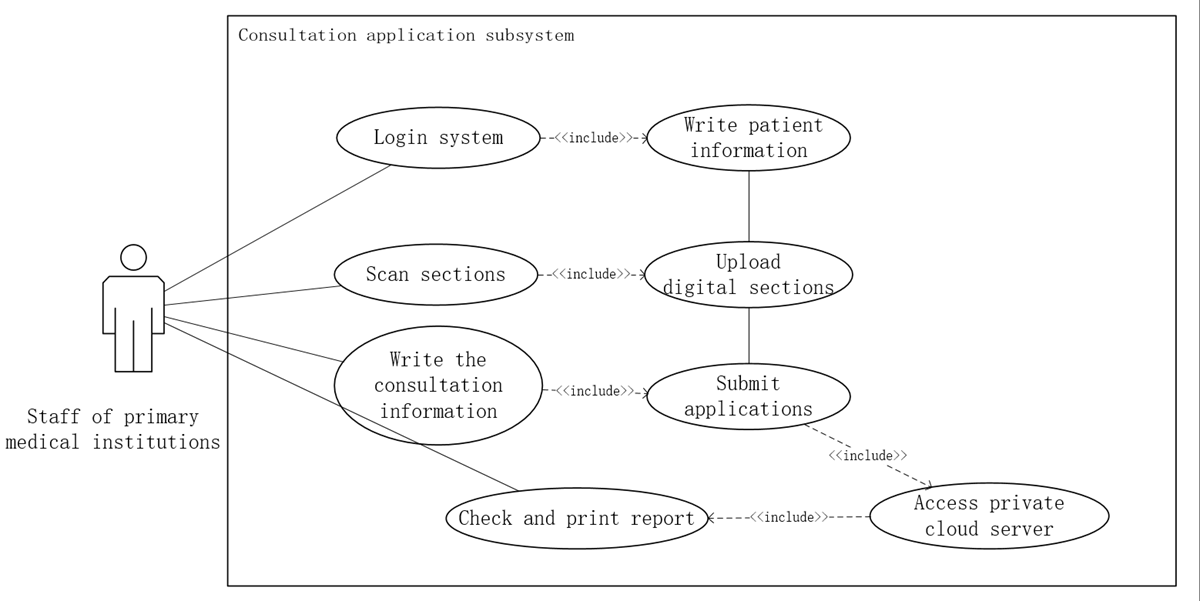
Workflow for telepathology consultation request submission in a province-wide telepathology network in Henan Province, China.

The initiation of a consultation application is a core function performed by requesting personnel at primary health care institutions. This process involves several key steps: logging into the system, scanning pathology slides, and completing the consultation application form. The output of this function is the transmission of consultation application data via the virtual private network (VPN) to the telemedical private cloud server at the Henan Telemedicine Center of China. This information is then made accessible to triage personnel and diagnostic experts at tertiary medical institutions for review and further processing. The use case diagram for this function is illustrated in Figure 5.

#### Triage Workflow and QC

The triage function is performed by pathologists serving as triage personnel within tertiary medical institutions, playing a pivotal role in ensuring the integrity of the diagnostic process. This function involves conducting preanalytical QC on received telepathology applications, which includes the following:

1. Assessing the adequacy of scanned WSIs for diagnostic interpretation, including sufficient tissue coverage, effective resolution, and focus across the tissue area, and absence of major artifacts (eg, excessive blur, stripe or banding, or large occlusions) that may compromise interpretation; staining or color issues were assessed in terms of image readability.
2. Verifying the completeness of clinical information, ensuring that relevant patient history, laboratory findings, and any necessary supporting documentation are provided to facilitate accurate diagnosis.

**Figure 6:**
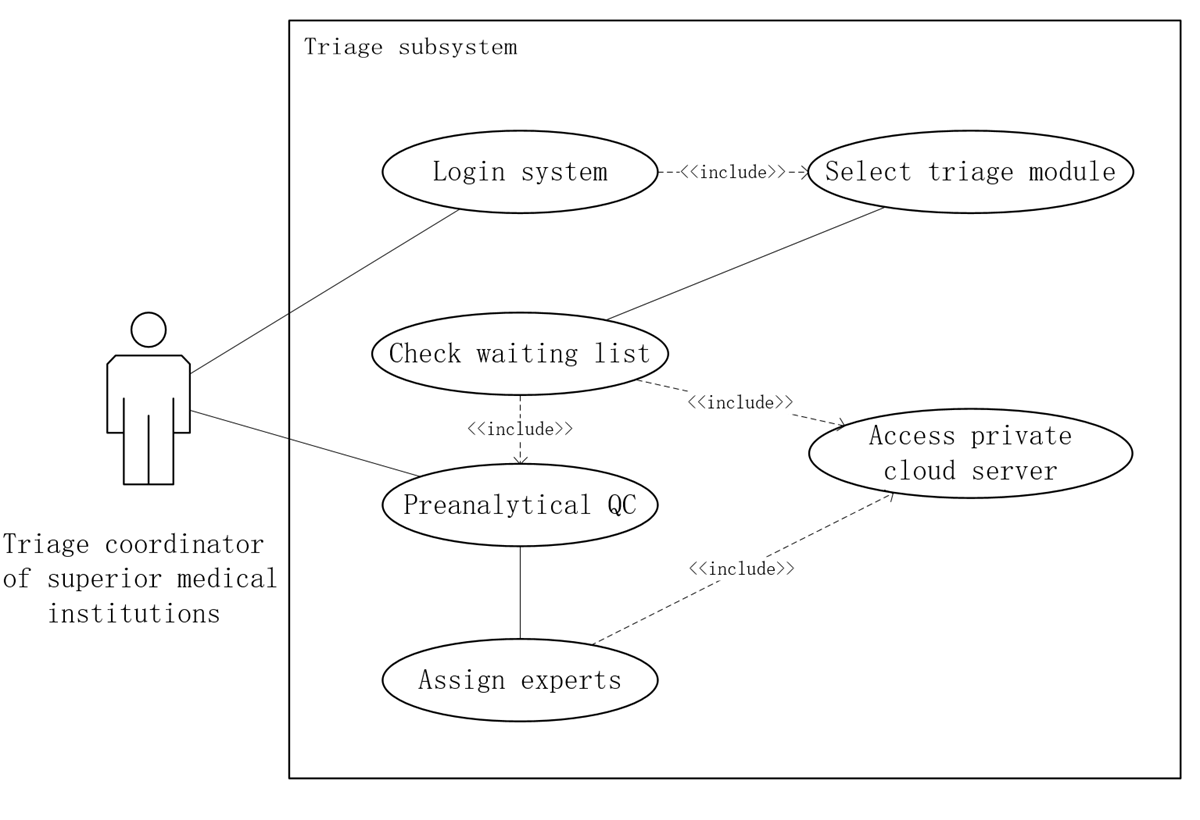
Workflow for case triage and preanalytical quality control in the province-wide telepathology system in Henan Province, China. QC: quality control.

If the WSI quality is insufficient for diagnostic interpretation or the clinical information is incomplete, triage personnel may request additional data or resubmission (eg, rescanning, recutting, or restaining when preparation-related issues are suspected) before assigning the case to the most appropriate diagnostic expert based on the specific pathology subspecialty required. The workflow diagram illustrating this process is depicted in Figure 6.

When resubmission was required, the case was returned within the platform with standardized reasons, and the requesting site was notified through in-platform status updates and SMS alerts to registered staff; unresolved returns were escalated to the site administrator for follow-up.

#### Diagnostic Function Design

**Figure 7:**
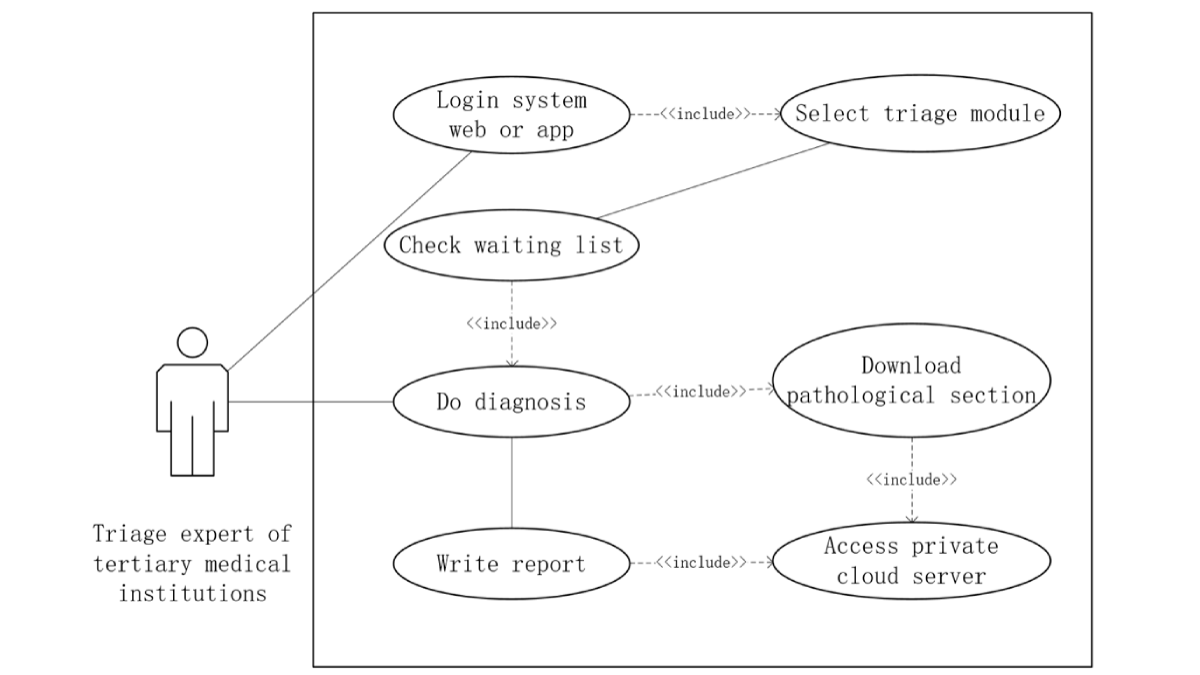
Workflow for remote pathology diagnosis and reporting within the province-wide telepathology system in Henan Province, China.

The diagnostic function is executed by experts at tertiary hospitals, specifically pathologists, who access the system via a computer interface or the WeChat mini-program. Upon logging in, they review the assigned telepathology cases, analyzing DP slides and accompanying clinical data to provide a comprehensive pathological diagnosis. The diagnostic report includes a pathological description, grading, staging, results of immunohistochemistry, and additional relevant findings (Figure 7).

When an expert at a tertiary hospital determines that additional immunohistochemistry staining is required, they can submit an immunohistochemistry request through the system, specifying the recommended immunohistochemistry markers for further testing. This request is automatically recorded in the system and made accessible to the originating hospital. The originating hospital performs the immunohistochemistry staining locally, digitizes the stained slides, and uploads them back to the original case record within the platform. The tertiary hospital expert then reviews the newly uploaded immunohistochemistry-stained slides, incorporating the findings into a supplementary diagnostic report to ensure a comprehensive pathology assessment. The diagnostic report is completed using a semistructured interface, where predefined sections (eg, “Microscopic Findings,” “Diagnosis,” and “Comments”) guide report writing, while allowing free-text input to maintain clinical flexibility.

### Ethical Considerations

This retrospective multicenter study was approved by the Ethics Committee of the First Affiliated Hospital of Zhengzhou University (approval number 2025-KY-1532-002). The requirement for informed consent was waived because the study involved secondary analysis of deidentified routine telepathology data without direct participant contact.

All data were anonymized prior to analysis, and no personally identifiable information was accessible to the research team. Data were stored on secure institutional servers with restricted access in accordance with applicable data protection regulations. No compensation was provided, as this study involved retrospective analysis of existing data. All procedures adhered to institutional policies and the principles of the Declaration of Helsinki.

## Results

### System Deployment and Operational Integration

The telepathology system was successfully deployed within the Henan Telemedicine Center's private cloud infrastructure, leveraging a secure VPN to facilitate encrypted data transmission across 120 integrated health care institutions at the provincial, municipal, and county levels. This architecture ensures robust data security, transmission stability, and interoperability, which are critical for maintaining diagnostic accuracy during remote consultations.

The deployment of DP scanners followed a stepwise approach to ensure systematic integration, validation, and training across hospitals. In 2015, the Henan Provincial Health Commission initiated a province-wide infrastructure assessment to evaluate hospital readiness for telepathology implementation. The procurement and phased installation of 120 scanners began in 2016, prioritizing tertiary hospitals first, followed by city and county-level hospitals, based on workload demand and digital infrastructure readiness. Pathologists and technicians were trained in scanner operation, QC, and troubleshooting. By 2017, scanners were fully integrated into routine workflows, with ongoing monitoring and performance optimization to maintain consistency in image acquisition.

**Figure 8:**
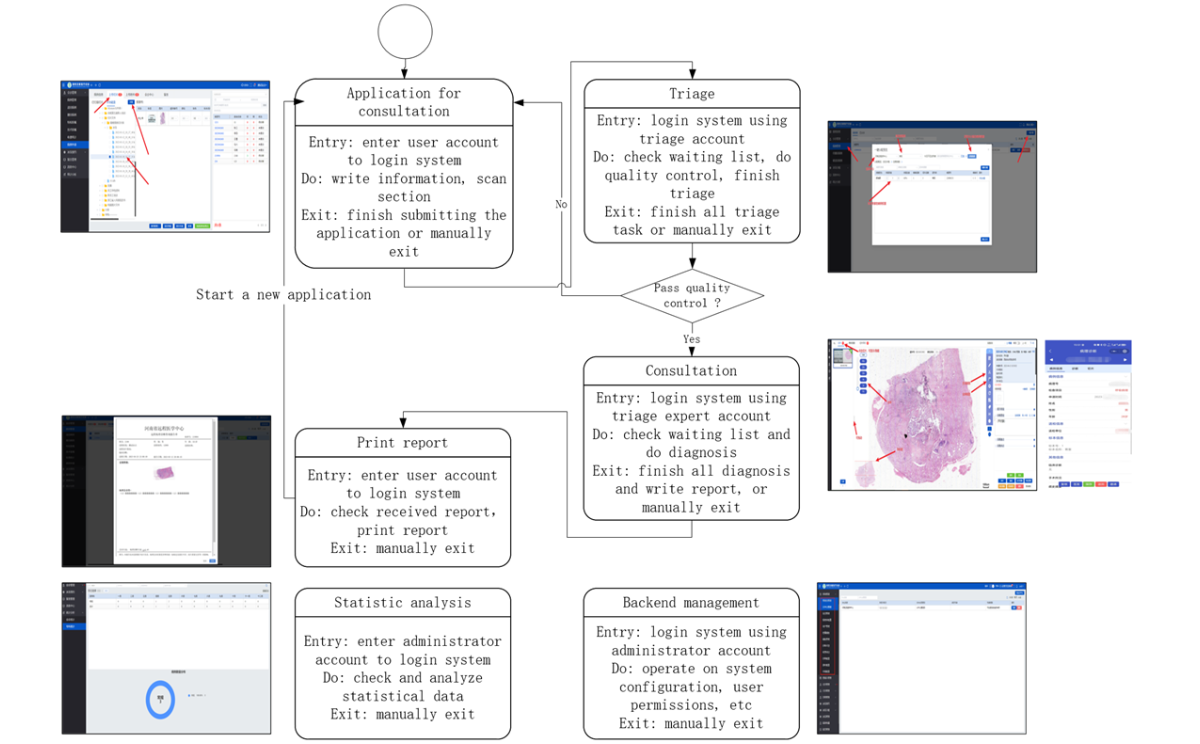
User interfaces of the web-based platform and WeChat mini-program used for telepathology consultations in Henan Province, China.

To accommodate various clinical scenarios, the system supports both web-based interfaces and mobile access via a WeChat mini-program. The mobile integration significantly enhances accessibility, particularly in rural and resource-limited regions, where mobile technology adoption is high. The user interface was optimized for both platforms to streamline diagnostic workflows, reduce user friction, and improve the TAT for pathology consultations (Figure 8).

Since deployment, the system has facilitated 72,916 telepathology consultations, averaging approximately 22 cases per day. A total of 355,104 WSIs have been processed. The cumulative data volume has reached 220.1 TB, with each slide averaging 0.62 GB. However, slide storage size varies depending on scanning resolution, typically ranging from 500 MB to 2 GB, with 20× scans occupying 500 MB to 1 GB and 40× scans ranging from 1 GB to 2 GB. The system supports multiple whole-slide image formats, including Tagged Image File Format (TIFF) and NDPI (NanoZoomer format), as well as other vendor-specific formats that use pyramidal storage for efficient multiresolution viewing. Unlike Digital Imaging and Communications in Medicine (DICOM), which is primarily used for radiology, these formats support multilayered, ultra-high-resolution whole-slide imaging, ensuring optimal fidelity for pathological assessment.

The system is hosted on Henan Telemedicine Center’s private cloud, using a distributed storage system for high availability, redundancy, and secure access. Diagnosed slides are archived rather than deleted, ensuring long-term availability for clinical review, QC, and research. To optimize storage, frequently accessed images remain in high-performance storage, while older slides are moved to cost-efficient archival storage, maintaining scalability as telepathology adoption grows.

### Diagnostic Process Implementation

Since deployment, the telepathology system has streamlined diagnostic workflows across 120 health care institutions, enabling seamless collaboration between county, city, and provincial-level hospitals. The system has facilitated the efficient submission, triage, and assignment of pathology cases, ensuring that cases requiring expert consultation are promptly routed.

The WeChat mini-program has been widely adopted to facilitate remote access and case review, allowing pathologists to view WSIs and provide feedback even outside traditional workstations. However, medical-grade monitors remain the preferred choice for primary diagnosis, while the mobile platform serves as a supplementary tool for preliminary assessments and report verification.

Health care professionals receive ongoing training and technical support to ensure effective system use. Annual training conferences provide updated guidance and case discussions. A comprehensive operations manual is available for reference, covering image acquisition, QC, and reporting workflows.

To optimize medical resource distribution, the system uses a tiered health care architecture, incorporating provincial, municipal, and county-level medical institutions. County-level institutions may escalate complex cases directly to provincial-level institutions or route them through municipal-level institutions. Municipal hospitals act both as requestors and providers, while provincial hospitals primarily manage complex case diagnostics.

### System Performance Evaluation

To evaluate the system’s technical performance, a stress test was conducted using the Apache Benchmark Tool. The detailed performance metrics are presented in Table 2. Simulated environments with 100 concurrent users performing 100 requests each were used to assess system responsiveness, scalability, and load-bearing capacity under both typical and peak usage conditions.

**Table 2:** Technical performance metrics of the province-wide telepathology platform under concurrent user load, evaluated using stress testing.

Requests	Response times (ms)						Throughput (transactions/s)		Network (KB/sec)	
Label	Min	Max	90th percentile	95th percentile	99th percentile			Received		sent
Consultation request interface	2568	7619	7013.10	7144.85	7497.85	25.78		114.72		48.81
Application list view page	190	3237	2859.50	2988.70	3206.30	58.88		646.77		30.24
Pending diagnosis list page	200	3017	2838.30	2891.75	3001.79	59.00		648.10		30.59
Diagnosis page	423	4359	3793.00	4031.70	4353.06	43.87		15.72		50.08

The data derived from the system performance evaluation reveals significant capabilities in handling concurrent operations across various interfaces:

1. Consultation request interface: the system can respond to 25.78 requests per second, which supports approximately 125 users operating online simultaneously.
2. Application list view page: the system is capable of handling 58.88 requests per second, allowing for about 300 users to be active online at the same time.
3. Pending diagnosis list page: the system’s response rate is 59 requests per second, indicating capacity for approximately 300 users to operate online simultaneously.
4. Diagnosis page: the system manages 43.87 requests per second, suggesting it can accommodate an estimated 220 users online simultaneously.

The results demonstrate that the telepathology system can maintain high performance even under substantial user loads. The integration of a dedicated VPN, coupled with the optimized Browser-Server architecture, ensures that diagnostic workflows are not compromised during peak operational periods. These capabilities are critical for large-scale deployments in health care systems with fluctuating demands.

### Real-World Impact and Clinical Outcomes

#### System Use and Expansion

The scanners deployed in hospitals were used for both routine in-house pathology workflows and telepathology consultations, but only selected cases were uploaded for remote expert review. The telepathology system was not intended to replace all local diagnostic workflows; rather, it served as a platform for second-opinion consultations and specialized expert input when necessary. Cases uploaded for telepathology consultations primarily included diagnostically complex cases requiring additional expert interpretation, subspecialist consultation requests for specific pathology types, and frozen section cases where rapid intraoperative decision-making was required. This selective approach ensured efficient use of DP resources while allowing hospitals to retain routine diagnostics locally. By integrating telepathology into the existing diagnostic framework, hospitals could leverage the expertise of higher-level institutions without overloading the digital infrastructure with routine cases that did not require external review.

Since its implementation, the telepathology system has been integrated into 120 health care institutions across Henan Province. From 2016 to 2024, a total of 72,916 diagnostic cases were completed, with municipal hospitals contributing 16,313 (22.37%) cases and county-level hospitals accounting for 56,603 (77.63%) cases. Provincial hospitals primarily participated as expert consultation centers and triage hubs; although technically able to submit cases, they rarely acted as requestors in routine practice. This hospital-level distribution was used as an indirect indicator of access to expert pathology support for lower-tier institutions. In China’s tiered health care system, county-level hospitals typically have more limited access to subspecialty pathology expertise than higher-level centers. Therefore, the predominance of county-level submissions indicates that the platform was primarily used by lower-tier institutions and facilitated routine access to tertiary-hospital expertise for remote sites.

Analysis of annual service volumes revealed a significant upward trend. Municipal hospitals experienced a growth from 240 cases in 2016 to 3503 cases in 2021, representing a 1360% increase. Similarly, county-level hospitals saw an increase from 2095 cases in 2016 to 8317 cases in 2019, reflecting a 297% growth. Despite fluctuations in service volumes in later years, the overall trend indicates sustained adoption and use across health care tiers (Table 1).

These findings suggest that the telepathology system has effectively enhanced diagnostic capacity, particularly in county-level institutions where pathology services were previously limited.

#### Diagnostic Efficiency and TAT

The system's efficiency was assessed based on TAT, defined as the interval from whole-slide image submission to diagnostic report issuance. The median TAT was 10.06 hours (IQR 1.63-29.10), with 54.28% (39,579/72,916) diagnosed within 12 hours and 96.41% (70,298/72,916) completed within 72 hours.

For frozen section consultations, where rapid diagnostic turnaround is critical, the system facilitated real-time notifications to pathologists upon case submission. From 2016 to 2024, a total of 508 frozen section cases were processed through the telepathology system, with an average time from request to diagnosis of approximately 16 minutes and 6 seconds. This highlights the system’s ability to support time-sensitive intraoperative pathology decisions and enhance surgical workflow efficiency.

#### Patient Demographics and Diagnostic Distribution

A total of 72,916 patient cases were analyzed. Patient age ranged from 2 to 91 years (mean 51.8, SD 16.9, median 52.6, IQR 45-67 years). Age-group distribution was as follows: 18 years, n=2187 (3%); 19-40 years, n=14,583 (20%); 41-60 years, n=38,652 (53.01%); and >60 years, n=17,494 (23.99%). Overall, 43.58% (n=31,777) of cases were male, and 56.42% (n=41,139) were female. Regarding diagnostic outcomes, 48.80% (35,583 cases) were benign, while 51.20% (37,333 cases) were malignant. Compared with a baseline dataset from 2020 [10], there was an observed increase in the proportion of male patients by 2.49% (from 41.09%) and a decrease in benign lesion diagnoses by 3.38% (from 52.18%).

The most frequently reported anatomical sites were uterine, gastrointestinal, and esophageal tissues. Other common specimen categories included breast, lung, mediastinum, and male genital organs. Additional sites included thyroid, head and neck, and respiratory tract. Although biopsy specimens were included, cases were categorized by anatomical site rather than procedural type (biopsy or resection), and detailed specimen-type classification was not available in the current platform; future updates may incorporate specimen-type fields to improve data granularity.

#### Economic Impact

Before the implementation of telepathology services, patients in county hospitals seeking expert pathology consultation typically had to travel to tertiary hospitals with their glass slides and medical records, incurring transportation costs, lost wages, and long wait times. Alternatively, some hospitals mailed slides for consultation, but this method was slow and inefficient, often leading to delayed diagnoses.

With telepathology, biopsy slides are now digitized locally and reviewed remotely by experts at tertiary institutions, eliminating the need for physical transport. Using the previously published per-case savings estimate of 378.5 RMB (approximately US $50) [10], and applying it to the annual consultation volumes observed in our network (Table 1), the estimated direct cost savings were approximately US $0.14 to $0.63 million per year across 2016-2024.

In the retrospective subset of 1027 consultations with paired diagnoses, the tertiary-hospital expert diagnosis recorded in the platform was treated as the reference standard (malignant vs nonmalignant). Originating-site preliminary diagnoses showed a sensitivity of 0.90 (481/535) and a specificity of 0.75 (369/492), with an overall agreement of 0.83 (850/1027). Overall, 177 (17.2%) cases were discordant, including 54 false negatives and 123 false positives (Table 3).

**Table 3:** Confusion matrix comparing originating-site preliminary diagnoses with tertiary-hospital expert reference diagnoses in telepathology consultations.

Originating-site preliminary diagnosis	Expert reference malignant (n=535), n	Expert reference nonmalignant (n=492), n
Originating-site malignant	481^a^	123^b^
Originating-site nonmalignant	54^c^	369^d^

^a^TP: true positive.

^b^FP: false positive.

^c^FN: false negative.

^d^TN: true negative.

## Discussion

### Principal Results

This study presents the design, deployment, and retrospective evaluation of a province-wide telepathology system implemented across multiple health care institutions in Henan, China. In line with the study aims, the system demonstrated (1) robust technical performance under concurrent load, (2) sustained real-world use with timely reporting, and (3) improved diagnostic accuracy in a retrospective subset compared with originating-site preliminary diagnoses. Overall, these findings suggest that a centrally coordinated telepathology network, supported by secure transmission and optional mobile access, can improve diagnostic access, operational efficiency, and quality assurance at scale.

### Interpretation and Implications

A key finding of this study was the high level of engagement from county-level hospitals. This pattern of use suggests that the telepathology network effectively expanded access to specialist pathology expertise for lower-tier and predominantly rural institutions, where in-house subspecialty support is often limited within China’s tiered health care system. By lowering geographic and institutional barriers, the system enabled routine consultation with tertiary-hospital experts and may help reduce long-standing disparities in diagnostic capacity.

From an operational perspective, the system supported timely reporting for routine consultations and rapid turnaround for time-sensitive frozen-section cases, indicating that telepathology can be integrated into both routine and intraoperative workflows without compromising clinical responsiveness. The availability of mobile access via a WeChat mini-program further enhanced flexibility for case review and report verification, particularly for clinicians working outside traditional workstations, while maintaining compatibility with established diagnostic practices.

Expert review conducted through the telepathology network corrected a meaningful proportion of preliminary diagnoses originating from lower-tier institutions. This finding underscores the role of telepathology not only in expanding access but also in strengthening diagnostic quality assurance by enabling second opinions and subspecialty input for complex or ambiguous cases.

In addition to clinical benefits, the system demonstrated potential economic value by reducing the need for patients to travel to tertiary hospitals for expert consultation. These cost-related benefits may be particularly important for rural populations, for whom travel expenses and delays can pose substantial barriers to timely diagnosis and care.

### Comparison With Prior Work

Prior studies have shown that telepathology can improve access to subspecialty expertise, shorten diagnostic turnaround, and support pathology services in resource-limited or geographically dispersed settings [19,23,24]. However, most reported implementations are confined to single institutions, pilot programs, or short-term projects. As a result, their impact on system-level diagnostic inequities remains limited [26].

In this context, this study differs in both scale and duration. We report a province-wide telepathology network involving 120 health care institutions across multiple levels of care, sustained over nearly a decade. Previous regional or academic center–based networks, including the California Telepathology Service, have demonstrated feasibility and operational benefits but were implemented within more restricted organizational or geographic boundaries [25,30]. By integrating telepathology into a tiered provincial health care system, our network primarily served county and city-level hospitals while using tertiary centers as expert hubs, a service and governance model that has been less frequently described.

Earlier work has emphasized that successful telepathology implementation relies on standardized image acquisition, secure data transmission, reliable storage, and structured quality assurance workflows [20,31,32]. The system evaluated in this study follows these principles through province-wide scanner deployment, centralized triage, and private-network transmission. At the same time, it addresses practical challenges related to large-scale WSIs storage and concurrent access, which have been identified as key barriers to broader adoption [26,28].

Remote access has become increasingly important for pathology workflows, particularly during periods of workforce shortage or limited on-site availability [33,34]. Most prior studies have focused on web-based or remote desktop solutions for diagnostic review. In our network, the WeChat mini-program was introduced as a complementary tool, supporting case monitoring, preliminary review, and report verification.

Regarding diagnostic quality, previous validation and consultation studies have reported that telepathology-supported second opinions can improve diagnostic confidence and help resolve challenging cases [19,25,34,35]. Our findings are consistent with this literature and indicate that expert review through the telepathology network corrected a substantial proportion of preliminary classifications. This supports the role of telepathology as a quality assurance and capacity building tool, rather than a replacement for local diagnostic workflows.

Economic analyses of telepathology have mainly focused on reductions in patient travel, slide transport, and associated delays [10]. The estimated cost savings in this study, derived by applying a previously published per-case savings estimate to observed consultation volumes, are consistent with prior reports and suggest that large-scale telepathology deployment may yield meaningful aggregate benefits for patients and health care systems. Nonetheless, as highlighted in prior studies, comprehensive cost-effectiveness evaluations that incorporate long-term outcomes and system-level resource use remain scarce and should be addressed in future research [4,26].

### Limitations

This study has several limitations. First, use and workflow metrics were derived from routinely collected platform data and reflect cases submitted for telepathology consultation rather than all pathology cases processed locally, which may limit representativeness. Second, diagnostic accuracy was assessed retrospectively in a subset of cases using expert diagnoses as the reference standard, and therefore does not capture interobserver variability or replace prospective multireader validation. Third, the economic impact was estimated rather than assessed through a formal cost-effectiveness analysis that incorporates long-term outcomes and system-level resource use. Specifically, we adopted a published per-case savings estimate for the same program [10] and extrapolated annual savings using observed consultation volumes, which may not capture variation in travel distance, local costs, or indirect costs. Finally, the study was conducted in a single province, and generalizability may vary in regions with different infrastructure and workforce distribution.

### Clinical Prospects

This province-wide implementation provides a practical blueprint for scaling telepathology in resource-limited settings by combining standardized workflows, secure transmission, and multiterminal access. Future work should include prospective validation across broader case mixes, formal economic evaluation, and continued investment in training and quality assurance to sustain diagnostic reliability across health care tiers. Incorporating AI-assisted decision support and interoperability standards may further enhance efficiency and consistency as adoption expands [36].

### Conclusions

A province-wide telepathology network can improve access to expert pathology services, support timely reporting, and enhance diagnostic performance in real-world practice. The high use of county-level hospitals suggests potential to reduce diagnostic inequity by extending specialist capacity to lower-tier institutions. This model may inform regional pathology capacity building and scalable telepathology deployment in other settings.

## Abbreviations

DICOM: Digital Imaging and Communications in Medicine
DP: digital pathology
QC: quality control
TAT: turnaround time
TIFF: tagged image file format
VPN: virtual private network
WSI: whole-slide image

## Data Availability

The study data are not publicly available because of legal, ethical, and privacy restrictions related to provincial health information regulations. The datasets generated or analyzed during this study are available from the corresponding author upon reasonable request.
